# Automatic segmentation and volumetric assessment of internal organs and fatty tissue: what are the benefits?

**DOI:** 10.1007/s10334-021-00986-1

**Published:** 2021-12-17

**Authors:** Fritz Schick

**Affiliations:** Department of Radiology, Section of Experimental Radiology, University Tübingen, Tuebingen, Germany

The most important goal of traditional medical imaging in the clinic is to show pathological changes on projection or slice images. The crucial criterion for the quality of the images, apart from spatial resolution, is usually the contrast between normal and pathologically changed tissue.

All currently available imaging modalities, including X-ray technology, ultrasound, magnetic resonance and positron emission tomography, have developed very rapidly over the past decades. Not only has the quality of the images improved while examination times have become shorter, but new aspects of the evaluation of the image data have also come into focus.

Particularly in the case of MRI, which in the past was often unable to exploit its full potential in the usual examination times of about 30–45 min due to relatively long measuring times for each imaging sequence, the modern methods with “massive parallel imaging”, [1] “compressed sensing” [2] and/or “finger printing” [3] are of benefit: it is now possible to perform comprehensive multi-parametric and quantitative tissue analyses in tolerable examination times. Instead of one “contrast weighting”, raw data for several quantifiable tissue properties (T1, T2, T2*, fat content, water diffusion, etc.) can now be collected in the same examination time. Furthermore, modern MRI systems allow the acquisition of high-resolution isotropic image data sets of the entire body or at least of the body trunk, which contain a large amount of morphological information.

The evaluation of anatomical–morphological information in 3D image data sets was initially limited to the manual determination of obvious disease-relevant information, e.g. for assessing the size of tumours or metastases in staging or for monitoring the course of disease under therapy [4]. While the (maximum) diameter of an anatomical structure could be determined rather quickly, it was much more time-consuming to perform volumetric analyses, as several slices had to be evaluated manually for this purpose. Somewhat delayed to the development of MR acquisition methods for high-resolution 3D datasets, automatic or at least semi-automatic segmentation methods were developed and used in many studies [5]. With the introduction of modern artificial intelligence (AI) methods, the possibilities for reliable organ segmentation from MRI datasets have improved significantly in recent years [6]. Only with the help of these methods has it now become feasible to evaluate individual anatomical structures in thousands of 3D data sets from large population based cohort studies (e.g., UK Biobank [7] or German National Cohort [8]) with manageable effort. With these data, on the one hand, correlations between anatomical features, genetic characteristics, life circumstances (e.g., nutrition and physical activity) and physical condition (physical fitness and diseases) can be explored. On the other hand, the results of volumetric assessment of organs in cohort studies are also very well suited for working out standard values and normal value ranges for different population groups depending on age and gender.

An important question is, of course, whether these volumetric data can also be used as indicators of disease in individual patients. The examination conditions (resting position, relatively uniform spatial sensitivity with modern multi-array head coils) and evaluation conditions (few tissue classes with relatively consistent spatial distribution) are quite favourable for MRI examinations of the brain. In addition, there are many institutions involved in neuroscience. Therefore, applications of volumetric assessment of brain structures [9] are certainly the most advanced and already well-studied for clinical questions [10].

The severity and time course of white and grey matter loss in different brain areas can provide differential diagnostic clues in diseases [11] and quantify the development of atrophy [12]. Deviations from normal age-related atrophy can be detected and effects of therapeutic interventions can be accurately measured.

For the work presented in many publications “home-made” segmentation algorithms have been applied, which are not easily applicable by the reader. Often the AI methods are trained with specific MRI images (in terms of contrast weighting, geometric imaging parameters, and coil arrangements) and a general applicability is not certain even if the algorithm is published. Recently, however, some MR vendors offer complete packages of sequences and segmentation tools that work reliably (e.g., for automatic segmentation of the liver). In neuro MRI studies, free software for segmentation (e.g., SPM, Wellcome Centre for Human Neuroimaging, UCL Neurology, London, UK) is available, which is used by many scientific groups and which can also be safely combined with clinical acquisition sequences. Furthermore, there is commercial support especially for post-processing of data from cohort studies from companies such as AMRA Medical (Linköping, Sweden).

To better assess the clinical relevance of automated segmentation and volumetric assessment of organ systems in the trunk of the body, some information about the significance of organ volume in diseases is provided in the following:

## Lungs

The individual lung volume is of course dependent on the breathing position and thus variable. Thus, consistent examination protocols are necessary for both reference and comparative measurements. Congenital diseases such as diaphragmatic hernias or bronchopulmonary malformations are often associated with a reduced lung volume. To obtain normal values in early childhood development, a number of lung volume examinations have been performed in foetuses depending on gestational age, but the results have not been entirely consistent [13]. In adults, acquired restrictive lung diseases such as pulmonary fibrosis or sarcoidosis can lead to reduced lung volume. In contrast, emphysema and COPD show overinflation of the lungs and thus often an increase in lung volume [14].

## Heart

**Fig. 1:**
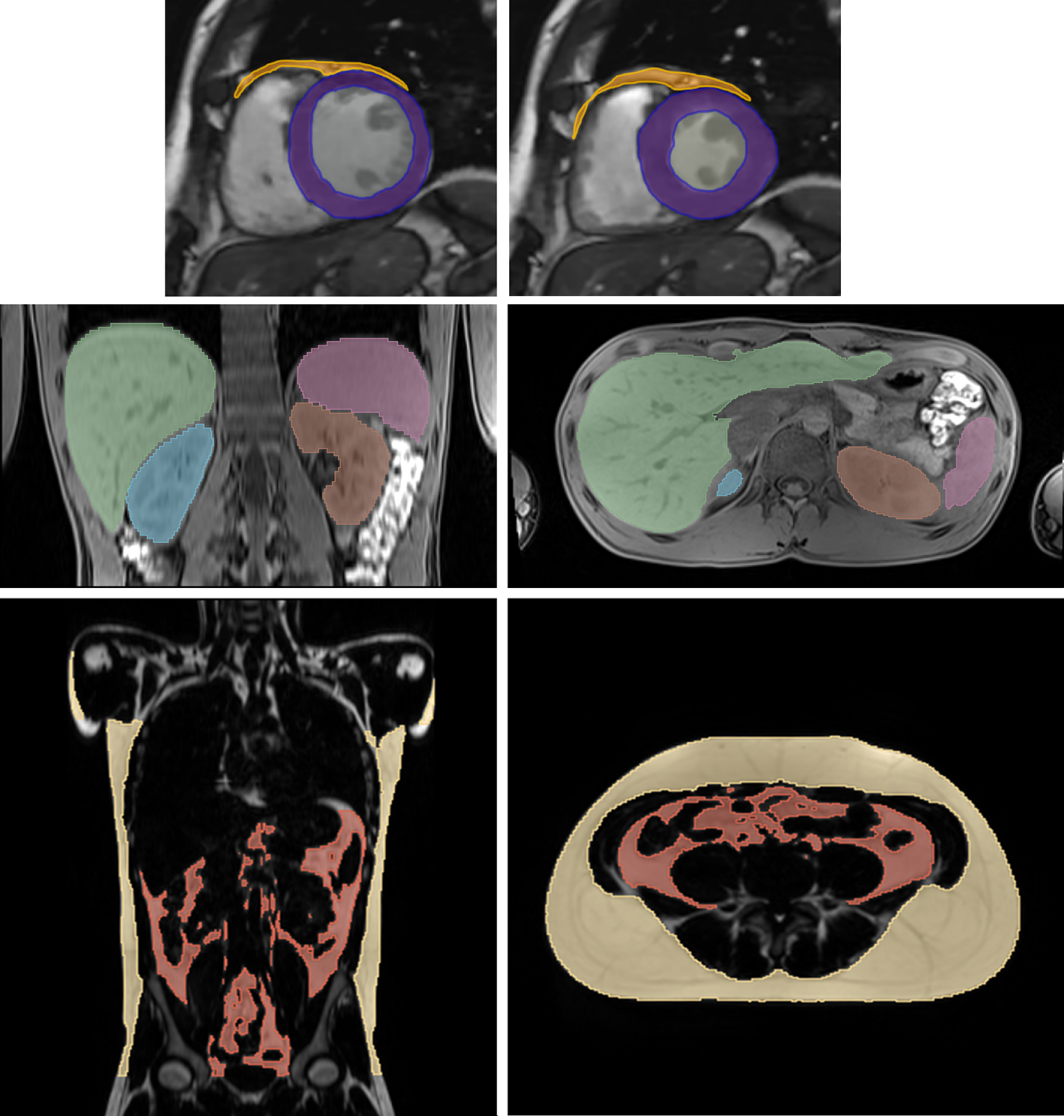
Shows in the top row two short-axis views of the heart in diastole (left) and systole (right). The myocardium of the left ventricle is segmented and coloured purple, areas of epi-/pericardial fat are depicted in orange. The middle row shows MR images with segmentation of internal organs as liver (green), spleen (light purple) and left (brown) and right (blue) kidneys. The two images in the bottom row highlight the subcutaneous fat (yellow) and visceral fat (red)

In cardiac patients (e.g., with dilatation or hypertrophy of the myocardium), MRI scans are performed with ECG synchronisation. Imaging in multiple cardiac phases allows assessment of the thickness of myocardial walls, systolic and diastolic blood volumes and derived functional parameters [15, 16] (see upper row of images in Fig. 1). The usual MRI data sets of the trunk are acquired without ECG triggering and are, therefore, usually not evaluable with regard to cardiac size and function. However, modern MRI procedures have already been described that can reconstruct respiratory and cardiac phase-dependent MRI images even without external triggering [17], as well as automated procedures for determining cardiac contours [18].

## Liver

In healthy liver, the organ volume can be considered a good parameter for the function of the organ. Liver size with normal value ranges has been investigated in CT studies, and formulas for “normal liver volume” as a function of body size have been given [19]. Those have been further refined and also adapted for different ethnicities [20, 21]. Parenchymal liver diseases such as steatosis, iron overload or cirrhosis are reported to significantly increase liver volume compared to normal values [21]. It should be noted, however, that the functionally active fraction of liver volume becomes smaller in cases with restrictive remodelling processes (hepatic fibrosis and cirrhosis) and must be determined separately by segmentation of the internal hepatic structures [22] Lifestyle changes in patients with steatosis led to a significant reduction in liver volume in parallel with a reduction in liver fat content [23, 24]. Particularly important clinical significance is given to the functional liver volume still available after partial resection (in the case of tumours or transplants), which must be taken into account when planning surgical procedures [25]

## Spleen

Enlarged spleen (splenomegaly) is often caused by liver disease associated with hypertension in the portal system. The relationship between the degree of liver fibrosis or portal hypertension and spleen volume has recently been quantitatively studied in a large number of patients [26]. Splenomegaly with often markedly increased volume compared to normal is also observed in some infectious diseases (viral diseases such as EBV and CMV; but also in malaria or toxoplasmosis) and in haematological (thalassaemia, sickle cell anaemia) or immunological (juvenile arthritis, lupus erythematosus) disorders [27].

## Pancreas

It has been reported that pancreatic volume tends to be reduced in both type I [28] and type 2 diabetes [29] compared with healthy individuals. In acute pancreatitis, an increased volume of the organ is observed [30], but after several acute episodes of pancreatitis have subsided, the pancreas volume is often significantly reduced [31]. Manual or automatic segmentation of the pancreas on CT or MR images in the case of (peripheral) fatty infiltration is hardly reliable [32]. Fatty infiltration of the pancreas is particularly common in cystic fibrosis, and differentiation from the surrounding fatty tissue is then often very difficult [33].

## Kidneys

In the kidney, the two main components, cortex and medulla, can usually be reliably segmented separately in 3D data sets from CT scans [34] and MRI scans [35]. The volume of the renal cortex in particular decreases significantly with age, while the volume of the renal medulla remains constant or even increases [34]. Deviations from normal values are seen on the one hand in congenital aplasia or hypoplasia of the kidneys, but also in acquired malfunctions. Enlarged, swollen kidneys occur in acute inflammatory processes (pyelonephritis) and in chronic diseases in early stages, while long-term kidney diseases (e.g., diabetic kidney) potentially cause fibrosis with a loss of function and a reduction in volume [36]. A disease pattern that is particularly frequently examined volumetrically using MRI is hereditary autosomal dominant polycystic kidney disease (ADPKD) [37].

## Adipose tissue (subcutaneous, visceral, epi- and pericardial)

Over the past 2 decades, many studies on the role of body fat in the development of metabolic and cardiovascular disease have been conducted. It has been demonstrated that it is not so much the total amount of adipose tissue as the distribution of adipose tissue in different compartments that plays an important role [38]. While subcutaneous adipose tissue contributes little to the development of metabolic-associated diseases, the amount of visceral fat located around abdominal organs, with its propensity for chronic inflammation, correlates significantly with reduced insulin action [39]. Larger amounts of visceral fat and also of fat in the thoracic cavity surrounding the myocardium (epicardial and pericardial fat, which are difficult to distinguish in MR images because they are separated by only a thin layer) have been identified as risk factors for cardiovascular disease [40]. It was also shown that smaller fat compartments such as neck fat are subject to independent regulation and that the amount of fat in the renal sinus area is related to proteinuria [41].

Since simple non-invasive methods such as body impedance measurement cannot determine some fat compartments such as visceral fat with sufficient accuracy, MRI with its ability to record volume data sets can also be employed excellently in this area.

## Skeletal musculature

A reduction in the quantity (and in some cases quality) of skeletal muscle is called sarcopenia. Especially in older patients, sarcopenia often leads to reduced activity and mobility and can result in loss of independence. Monitoring muscle build-up also plays a role in training after immobilisation due to illness or in sports medicine. Depending on the region of interest, cross-sectional areas of muscles or entire volumes can be quantitatively assessed by CT or MRI [42].

## Discussion

MRI is very well suited to quantitatively investigate anatomical structures in high-resolution 3D image datasets, both in terms of their size and volume (Fig. 1) as well as their signal properties. The alternative method CT is unfortunately associated with a radiation exposure that can hardly be justified when examining volunteers, and 3D ultrasound examinations are currently not readily possible for all anatomic structures in the body trunk, since air and bone-containing areas are not penetrable for ultrasound and the tissue contrasts are often critical for automatic evaluation.

As MRI is generally not associated with harmful effects on tissue, this method is also particularly well suited for examinations of large, mainly healthy cohorts, although this is relatively cost-intensive. The determination of organ volumes and fat volumes in large cohorts with healthy individuals from different age groups and genders allows the determination of normal value ranges, so that results of later individual examinations can be better assessed. However, systematic errors due to the use of different recording or image analysis methods must be taken into account.

In large cohorts from the entire population, individuals with diseases and/or medications are included, so that previously unknown correlations can also be recognised and further elucidated (in analogy to work on the brain as [43]). This allows new pathomechanisms to be detected and existing hypotheses to be tested. Since random correlations sometimes occur with a large number of parameters under investigation, careful statistical analysis (with Bonferroni correction) and the verification of results in independent collectives are indispensable.

## Individual studies

The question arises: is every size specification of an organ or its substructures relevant? For most questions this is probably not the case, at least as long as the measured values are in the “normal range”. Significant deviations from the normal range, however, can indicate pathological processes as reported above or confirm already existing suspected diagnoses. It should also be noted that experienced radiologists will often notice significant deviations of the organ size from the normal range when reviewing the images, even without automated assessment.

Visceral and epi-/pericardial fat volumes in particular, along with other conditions, are considered risk factors for the development of diabetes and cardiovascular disease. A determination of the volume as well as therapeutic measures to reduce those fat compartments is, therefore, useful. Individual measurement of fat volume reduction is often very helpful in motivating patients when implementing appropriate therapies; if, for example, muscle mass increases and fat mass decreases during a lifestyle intervention with more physical activity, this is not visible through simple weight or whole body impedance measurements. In those cases, corresponding efforts are discontinued if no positive encouragement is given through a demonstrable success. The same holds true for monitoring the strengthening of the skeletal muscles through training after immobilisation due to illness or in the field of sports.

With the rapid development of automatic segmentation methods using IA algorithms, it is quite conceivable that MRI (and possibly clinically indicated CT) examinations of the body trunk will in future allow organ volumes and other quantitative measurements to be determined automatically. Similar to the routine laboratory chemical analysis of blood and other body fluids, deviations from the normal range will then be indicated and can be exploited to make a diagnosis.
